# Ferromagnetic-like behavior of Bi_0.9_La_0.1_FeO_3_–KBr nanocomposites

**DOI:** 10.1038/s41598-019-46834-0

**Published:** 2019-07-18

**Authors:** Dmitry V. Karpinsky, Olena M. Fesenko, Maxim V. Silibin, Sergei V. Dubkov, Mykola Chaika, Andrii Yaremkevich, Anna Lukowiak, Yuri Gerasymchuk, Wiesław Stręk, Andrius Pakalniškis, Ramunas Skaudzius, Aivaras Kareiva, Yevhen M. Fomichov, Vladimir V. Shvartsman, Sergei V. Kalinin, Nicholas V. Morozovsky, Anna N. Morozovska

**Affiliations:** 1 Scientific-Practical Materials Research Centre of NAS of Belarus, 220072 Minsk, Belarus; 2 Institute of Physics, NAS of Ukraine, 46, pr. Nauky, 03028 Kyiv, Ukraine; 3 National Research University of Electronic Technology “MIET”, 124498 Moscow, Russia; 4 Institute for Bionic Technologies and Engineering, I.M. Sechenov First Moscow State Medical University, Moscow, 119991 Russia; 5 Institute of Low Temperature and Structure Research, PAS, Wroclaw, 50-422 Poland; 6 Institute of Chemistry, Vilnius University, Naugarduko 24, Vilnius, LT-03225 Lithuania; 7 Charles University in Prague, Faculty of Mathematics and Physics, V Holešovičkach 2, Prague 8, 18000 Czech Republic; 8 Institute for Materials Science and Center for Nanointegration Duisburg-Essen (CENIDE), University of Duisburg-Essen, 45141 Essen, Germany; 9 Center for Nanophase Materials Sciences, Oak Ridge National Laboratory, Oak Ridge, TN 37831 United States

**Keywords:** Magnetic properties and materials, Nanoscale materials, Information theory and computation

## Abstract

We studied magnetostatic response of the Bi_0.9_La_0.1_FeO_3_– KBr composites (BLFO-KBr) consisting of nanosized (≈100 nm) ferrite Bi_0.9_La_0.1_FeO_3_ (BLFO) conjugated with fine grinded ionic conducting KBr. When the fraction of KBr is rather small (less than 15 wt%) the magnetic response of the composite is very weak and similar to that observed for the BLFO (pure KBr matrix without Bi_1-x_La_x_FeO_3_ has no magnetic response as anticipated). However, when the fraction of KBr increases above 15%, the magnetic response of the composite changes substantially and the field dependence of magnetization reveals ferromagnetic-like hysteresis loop with a remanent magnetization about 0.14 emu/g and coercive field about 1.8 Tesla (at room temperature). Nothing similar to the ferromagnetic-like hysteresis loop can be observed in Bi_1-z_La_z_FeO_3_ ceramics with z ≤ 0.15, which magnetization quasi-linearly increases with magnetic field. Different physical mechanisms were considered to explain the unusual experimental results for BLFO-KBr nanocomposites, but only those among them, which are highly sensitive to the interaction of antiferromagnetic Bi_0.9_La_0.1_FeO_3_ with ionic conductor KBr, can be relevant.

## Introduction

## Magnetic, structural and electrophysical properties of pristine BiFeO_3_

Multiferroics are ideal systems for fundamental studies of couplings among the order parameters of different nature, e.g. ferroelectric (**FE**) polarization, structural rotational antiferrodistortion (**AFD**), ferromagnetic (**FM**) and antiferromagnetic (**AFM**) long-range order parameters^1–6^. Magnetoelectric (**ME**) coupling is especially important for the most of multiferroic fundamental studies and applications^1–5^.

Bismuth ferrite BiFeO_3_ (**BFO**) is the unique multiferroic with the large FE polarization (more than 60 μC/cm^2^ at RT) and AFM order coexisting up to room (RT) and elevated temperatures^7,8^. BFO exhibits unusual electrophysical properties, such as conduction and magnetotransport enhancement at domain walls^9–15^. Specifically, bulk BFO exhibits AFD long-range order at temperatures below 1200 K; it is FE below Curie temperature *T*_*C*_ ≈ 1145 K and is AFM below Neel temperature *T*_*N*_ ≈ 645 K^16^. It is known that pristine bulk BFO is characterized by a cycloidal modulation of the magnetization superimposed on the AFM G-type magnetic structure^3^. Although the linear magnetoelectric effect is symmetry forbidden on a macroscopic scale, the Dzyaloshinskii-Moriya mechanism can be used locally in BFO to achieve an electric switching of the spin cycloid^17–20^.

## Magnetic, structural and electrophysical properties of Bi_1-z_La_z_FeO_3_

Chemical doping of pristine BFO with lanthanum La ions having ionic radius (1.16 Å^21^) similar to that of bismuth Bi ions (1.17 Å^20^) causes a structural transition from the polar rhombohedral phase to the anti-polar orthorhombic phase which is accompanied with a slight decrease in the unit cell volume^22–24^. The structural transition is associated with significant changes in the unit cell parameters, namely, the modification of the Fe–O chemical bond lengths (a shortening of the bond length between the iron ions and apical oxygen ions and an elongation of the chemical bond between the iron ions and the oxygen ions located in the basal a*b* plane), an increase in the Fe–O–Fe angles, decrease in the oxygen octahedra rotations and tilts etc. The mentioned changes of the structural parameters lead to a reduction of the polar displacement of the ions and to significant change of the magnetic properties of the compounds near the rhombohedral-orthorhombic phase boundary. The diffraction data obtained at RT for the compounds Bi_1-z_La_z_FeO_3_ (**BLFO**) indicate a single phase rhombohedral state for the compounds with z < 0.16, two phase structural state assuming a coexistence of the polar rhombohedral and the anti-polar orthorhombic phases in the concentration range 0.16 < z < 0.19; further increase in the dopant content leads to a stabilization of single phase orthorhombic state (incommensurately modulated anti-polar orthorhombic phase, anti-polar or non-polar orthorhombic phase) depending on the La content as described elsewhere^22,25,26^.

Sol-gel synthesis method used to prepare BLFO solid solution leads to a formation of oxygen stoichiometric compounds those magnetic properties are governed by the exchange interactions between Fe ions being in 3+ oxidation state. Increase in the concentration of the lanthanum La ions leads to a gradual reduction in the critical magnetic field associated with a disruption of the spatially modulated spin structure and the compound with lanthanum content z ~ 0.17 is characterized by weak ferromagnetic structure at RT assuming complete disruption of the spatially modulated structure. The structural transition from the rhombohedral to the orthorhombic phase is accompanied by a significant increase of the remanent magnetization and coercive field, which is observed for the compounds having dominant or single phase orthorhombic structure.

It should be noted that transport properties of the BLFO compounds are also strongly dependent on the La concentration, and the conductivity of these solid solutions gradually increases with the dopant content increase up to z = 0.15^27^. Impedance measurements have shown mainly p-type conductivity of the compounds having single phase rhombohedral structure which significantly increases with temperature^22^.

This work studies magnetostatic response of the newly synthesized Bi_0.9_La_0.1_FeO_3_ - KBr composites (BLFO-KBr) consisting of nanosized (≈100 nm) ferrite Bi_0.9_La_0.1_FeO_3_ (BLFO) conjugated with fine grinded ionic conducting KBr. We revealed that when the fraction of KBr increases above 15%, it demonstrates ferromagnetic-like hysteresis loop with a remanent magnetization about 0.14 emu/g and coercive field about 1.8 Tesla (at room temperature). To the best of our knowledge nothing similar was reported previously in the literature. Different physical mechanisms were considered to explain the unusual experimental results for BLFO-KBr nanocomposites. The original part of the manuscript is structured as follows. Magnetostatic and electrophysical properties of BLFO–KBr nano-composites are discussed in **section II**. Theoretical explanation of the ferromagetism in BLFO–KBr nano-composites is given in **section III**. **Section IV** is a brief summary. Auxiliary experimental results and samples characterization are given in the Supplement.

## Magnetic and Electrophysical Properties of BLFO–KBr Nano-Composite

### Nanocomposite preparation and characterization

The samples of nanograined BLFO ceramics have been prepared by the aqueous sol-gel method using hydrates Bi(NO_3_)_3_·5H_2_O, Fe(NO_3_)_3_·9H_2_O and lanthanum nitrate as starting materials. The prepared sol-gel samples were heated at 800 °C for 1.5 h^28,29^. The composites have been prepared by thorough mechanical mixing of the single phase ferrite (Bi_0.9_La_0.1_FeO_3_) and grinded KBr taken in the next mass ratios – 90:10, 85:15, 80:20, 70:30, 50:50 respectively. The obtained powders have been uniaxially pressed at compacting pressures of 5 GPa to obtain tablets with 5 mm diameter and 1 mm thickness.

**Figure 1:**
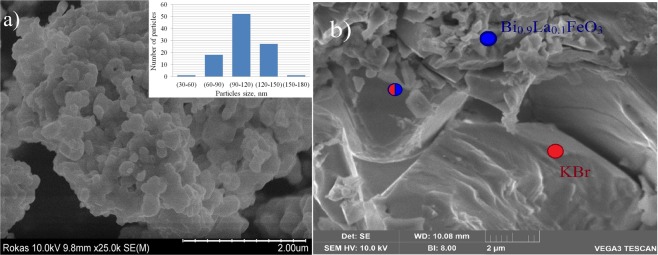
(**a**) SEM image of the compound Bi_0.9_La_0.1_FeO_3_ obtained at room temperature. The inset shows a distribution of crystalline size over the SEM image of the compound. (**b**) SEM image of the composite 50% Bi_0.9_La_0.1_FeO_3_ −50% KBr, the spots marked by different colors denote the areas attributed to the ferrite phase, KBr phase or their mixture as determined by EDS measurements (detailed results of EDS experiments are presented in the Supplement).

The compound Bi_0.9_La_0.1_FeO_3_, which has been mainly used to prepare the composites, is characterized by the grains with average radius of about 100 nm, and the small amount of the grains have the average radius of about 35–40 nm (the image obtained by scanning electron microscopy (SEM) and the histogram of the grain sizes distribution are shown in the Fig. 1(a). SEM measurements performed for the composites have allowed to clarify their morphology and to specify a distribution of the constituent phases [Fig. 1(b)]. The crystallines having micrometer size are attributed to the KBr phase as determined by EDS analysis carried out for the selected areas of the SEM images [see Supplement, Fig. S1]. Elemental composition of the constituent components of the composites is confirmed by EDS data presented in the Supplement.

Characteristic size of the grains attributed to KBr phase is in the range of 0.100–10 μm. The crystallines ascribed to the ferrite phase are characterized by rectangular-shaped form, ferrite grains are mainly agglomerated into clusters having size of about 1–2 microns, while separate grains are characterized by nanometer size as confirmed by the SEM images obtained for the composites and the single phase ferrite [compare Fig. 1(a) and 1(b)]. The SEM data have confirmed homogeneous distribution of the constituent phases in the composites which facilitates active chemical processes in the boundary region of both phases.

The modified sol-gel synthesis method and preparation conditions^28,29^ used to synthesize nanoscale powder of Bi_1-z_La_z_FeO_3_ (z = 0, 0.05, 0.1) lead to a formation of single phase stoichiometric ferrites utilized to form the composites. The X-ray diffraction measurements performed for the ferrite compounds have confirmed their single phase rhombohedral structure, the XRD patterns recorded at room temperature have been successfully refined assuming non-centrosymmetric rhombohedral space group *R3c*.

Under standard conditions, potassium bromide (KBr) is a white nonmagnetic crystalline powder which exhibits high ionic conductivity. Phase purity of the commercial KBr powder has been confirmed by the XRD measurements performed by the authors, the obtained results are in accordance with the structural data provided by the producer (see Supplement, Fig. S2). The XRD patterns obtained for the composites have confirmed a coexistence of two constituents having the phase ratios in accordance with the mentioned chemical formulas. The diffraction peaks attributed to the constituents do not show any modification of the reflections positions, their width, asymmetry etc. as compared to the reflections observed on the diffraction patterns obtained separately for BLFO and KBr (see Supplement, Fig. S2). Phase purity of the constituents assumes that the magnetic properties of the composites are determined only by the ferrite component.

Under standard conditions, potassium bromide (KBr) is a white nonmagnetic crystalline powder which exhibits high ionic conductivity. Phase purity of the commercial KBr powder has been confirmed by the XRD measurements performed by the authors, the obtained results are in accordance with the structural data provided by the producer (see Supplement, Fig. S2). The XRD patterns obtained for the composites have confirmed a coexistence of two constituents having the phase ratios in accordance with the mentioned chemical formulas. The diffraction peaks attributed to the constituents do not show any modification of the reflections positions, their width, asymmetry etc. as compared to the reflections observed on the diffraction patterns obtained separately for BLFO and KBr (see Supplement, Fig. S2). Phase purity of the constituents assumes that the magnetic properties of the composites are determined only by the ferrite component.

For comparison with the composite, the temperature dependences of magnetization for the nanograined ferrites Bi_1-z_La_z_FeO_3_ (z = 0, 0.05 and 0.1) have been measured in the temperature range 300–1000 K using MPMS SQUID VSM magnetometer (Supplement, Fig. S3). The isothermal magnetization measurements have been done at room temperature and at T = 5 K (Supplement, Fig. S4). The obtained results demonstrate a quasi-linear behavior of the magnetization dependencies (see Supplement, Fig. S4). An increase of the magnetization observed for the compounds with the dopant content up to ~13% in strong magnetic fields (5–8 Tesla) is associated with a partial disruption of the spatially modulated magnetic structure, the transition is reversible and after exposure to magnetic field the magnetization of the compounds returns to nearly nullified values^30,31^. The very small values of the remanent magnetization (0.015–0.02 emu/g) observed for the lightly-doped compounds mainly depends on the dopant content and is caused by uncompensated spin magnetic moments formed in the surface layer of the crystallites due to disruption of the modulated magnetic structure. The compounds Bi_1-z_La_z_FeO_3_ with the dopant content z ≥ 0.15 are characterized by the anti-polar orthorhombic structure and have remanent magnetization of ~0.15–0.2 emu/g^31^. The compound Bi_0.9_La_0.1_FeO_3_ having rhombohedral crystal structure and modulated magnetic structure with almost zero remanent magnetization has been selected as the most appropriate compound for formation of the composites to estimate an influence of the constituents on the physical properties of nanocomposites.

### Magnetic and electrophysical properties of (Bi_0.9_La_0.1_FeO_3_)_x_–(KBr)_1-x_ nano-composites

**Figure 2:**
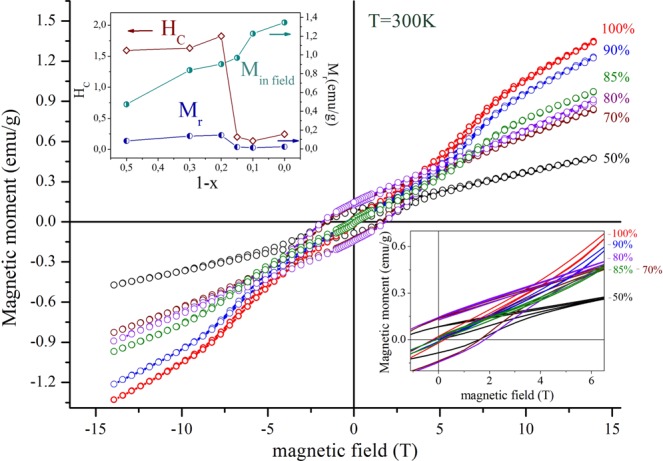
Dependencies of magnetic moment versus applied magnetic field for the composites (BLFO)_x_ – (KBr)_1-x_ at RT (the data for composites with the fractions of x = 100% are denoted by red symbols, 90% - blue, 85% - green, 80% - violet, 70% - brown and 50% - by black symbols. Left inset shows KBr fraction (1-x)-dependencies of M_r_, M_m_ and H_c_ for the composites; right inset shows enlarged part of the M(H) dependences.

The isothermal dependencies of magnetization (M) on magnetic field (H) of the nanocomposites (Bi_0.9_La_0.1_FeO_3_)_x_ - (KBr)_1-x_ (shortly (**BLFO**)_**x**_-(**KBr**)_**1**-**x**_) are shown in Fig. 2 for the fractions x = 100% (red symbols), 90% (blue), 85% (green), 80% (violet), 70% (brown) and 50% (black). The M-H-dependencies have been obtained in the fields up to 14 T using a magnetometer from Cryogenic Ltd. We have also confirmed experimentally that KBr matrix without ferrite compound has no magnetic response. Figure 2, left inset shows KBr-fraction (1-x)-dependencies of the remanent magnetization (**M**_**r**_), maximal magnetization (**M**_**m**_) at the maximal field *H*_m_ = 14 T and the coercive field (**H**_**c**_) for (BLFO)_x_-(KBr)_1-x_ composites.

The M(H) dependencies of the composites with the fraction of KBr less than 15% are similar to those observed for the compounds without KBr content (see red, blue and green symbols in Fig. 2). Further increase in the KBr fraction significantly changes the magnetic properties of the composites (BLFO)_x_–(KBr)_1-x_, namely leads to the appearance of ferromagnetic-like hysteresis of M(H) loops (see violet, brown and black symbols in Fig. 2). Note that the increase of the KBr fraction (1-x) above 15% leads to the step-like decrease of M_m_, and increase of M_r_ and H_c_ values of the composites (see Fig. 2 insets). The composite containing 20% of KBr is characterized by M_r_ ≈ 0.15 emu/g and H_c_ ≈ 1.8 T at RT (violet symbols in Fig. 2 and inset). Further increasing in the KBr fraction leads to the gradual decrease of M_r_, which is mainly associated with a dilution of the magnetically active ferrite component, wherein H_c_ attributed to the composites remains nearly unchanged (see violet, brown and black symbols in Fig. 2 and inset). The composite with equal fractions of the constituents (x = 50%) is characterized by the opened magnetization loop specific for ferromagnetic-like materials. Magnetization measurements performed at temperature ~5 K do not reveal any notable changes of the composites’ magnetic properties, slight increase in magnetization observed at temperature of ~5 K can be justified by a reinforcement of the magnetic interactions occurred at low temperatures.

Different physical mechanisms can be responsible for the appearance of ferromagnetic-like response of (BLFO)_x_–(KBr)_1-x_ nanocomposites, where BLFO with La content less than 16% is purely antiferromagnetic in the bulk at temperatures lower than 650 K and KBr is magnetically inactive material. Anyway, the changes observed in the magnetization behavior of the composites can be completely attributed to the changes occurred in the ferrite component. One of the most plausible models, describing the changes of the magnetic properties observed for the composites (BLFO)_x_–(KBr)_1-x_ depending on the KBr content assumes intensive chemical processes in the vicinity of ferrite particle - salt interface occurred during the composites formation. As a result of the redox reactions the Fe ions near the complex oxide – alkali halide interface can partially change their effective oxidation state from 3+ down to 2+. An uncompensated magnetic moment formed due to a difference between the magnetic moments attributed to the Fe^3+^ and Fe^2+^ ions within the antiferromagnetic matrix should lead to a notable increase of remanent magnetization of the composites and to a decrease of coercivity. Remanent magnetization estimated for the composites does not significantly change with an increase of KBr and it remains nearly stable for the composites with KBr fraction of about (20–50)%, which can be explained assuming that the chemical processes occur only in the thin sub-surface layer of the crystallites of the ferrite component. An average size of the ferrite crystallites is about 100 nm as confirmed by scanning electron microscopy measurements (see Fig. 1), that causes the presence of a concentration threshold (~20%) affecting the chemical processes and the composites having smaller KBr fraction does not show significant difference in magnetic and transport properties as compared to the ferrite compounds (see Supplement, Fig. S4). The manifestation of this effect might be a threshold of about ~20% of KBr content. This idea is verified by the isothermal magnetization curves obtained for the composites with KBr phase ratios 15% and 10% which have revealed almost absence of the remanent magnetization, while distinct opened hysteresis loop is observed for the composite having 20% of KBr content, so pointing at certain threshold. Notably, that the single-crystalline BiFeO_3_ powder synthesized by using molten KCl-KBr salt at 750 °C showed a very weak ferrimagnetic nature at low magnetic field^32^.

In order to get more information concerning the effect of KBr matrix we have measured the electroresistivity of the nanocomposites (BLFO)_x_-(KBr)_1-x_ and nanograined ceramics BLFO produced by the sol-gel method. Current-voltage (I-V) characteristics of all these samples are quasi-linear (see Supplement, Fig. S5), and the values of the electro-resistivity determined from I-V characteristics are listed in Table 1. The I (V) curves and resistivity values presented in the Table 1 demonstrate the pronounced decrease of electroresistivity with an increase of KBr content. Since the I(V) curves represent only ***dc*** electronic conductance it is not possible to itemize a contribution of the ferrite and KBr phases into the conductivity of the composites; *dc* conductivity of KBr phase is caused by a number of defects formed within the KBr grains, while an increase in the conductivity of the ferrite phase can be induced by a formation of Fe^2+^ ions in the surface layer of the ferrite grains due to redox reactions occurred because of the KBr constituent. It should be noted that a formation of a significant amount of the Fe^2+^ ions should lead to a rapid decrease of coercivity of the composites^33^ while the isothermal magnetization data (Fig. 2) do not show any notable decrease of coercivity.

**Table 1:** Electro-resistivity the nanocomposite _x_(BLFO)–_(1-x)_KBr.

	Composite _x_Bi_0.9_La_0.1_FeO_3_–_(1-x)_KBr				
Resistivity (in 10^9^ Ω/cm)	x = 1	x = 0.9	x = 0.7	x = 0.5	x = 0
	>10	5	2	0.1	<0.01

## Theoretical Explanation

### Discussion of the possible mechanisms

Different physical mechanisms (analyzed in the next subsection) can be responsible for the appearance of room-temperature weak ferromagnetism of Bi_1-z_La_z_FeO_3_ nanosized inclusions. Recently we constructed a comprehensive Landau-Ginzburg-Devonshire (**LGD**) thermodynamic potential and using it modelled the phase diagram of pristine BFO^34^. The role of the AFD, rotomagnetic (**RM**), and rotoelectric (**RE**) couplings was established in ref.^35^. This in complex allows reconstructing the phase diagram of BFO and long-range order parameter distributions including the temperature stability of the AFM, FE, and AFD phases, as well as prediction of novel intermediate structural phases.

Actually, Bi_1-z_La_z_FeO_3_ can exhibit weak ferromagnetic properties near the surface of micro- and especially nanoparticles via structural distortions unlocing the cycloid modulated spin structure and resulting in Fe^3+^ spins canting due to non-vanishing Dzyaloshinskii-Moriya (DM) interaction^17,18,36–40^. Different physical mechanisms can be responsible for the appearance of room-temperature ferromagnetism of ferroelectric Bi_1-z_La_z_FeO_3_ inclusions, while Bi_1-z_La_z_FeO_3_ is FE and purely AFM in the bulk^3^. Such mechanisms, in particular, are surface piezomagnetic effect existing near the surface of any antiferromagnetic due to the absence of spatial inversion center at the surface^41^, flexo-magnetoelectric^42^ and linear antiferrodistortive-antiferromagnetic^43^ couplings, Vegard effect (so called “chemical pressure”)^44,45^ as well as omnipresent magnetic defects and/or impurities accumulation at the surface due to the strong lowering of their formation energy at the surface^46,47^.

**Figure 3:**
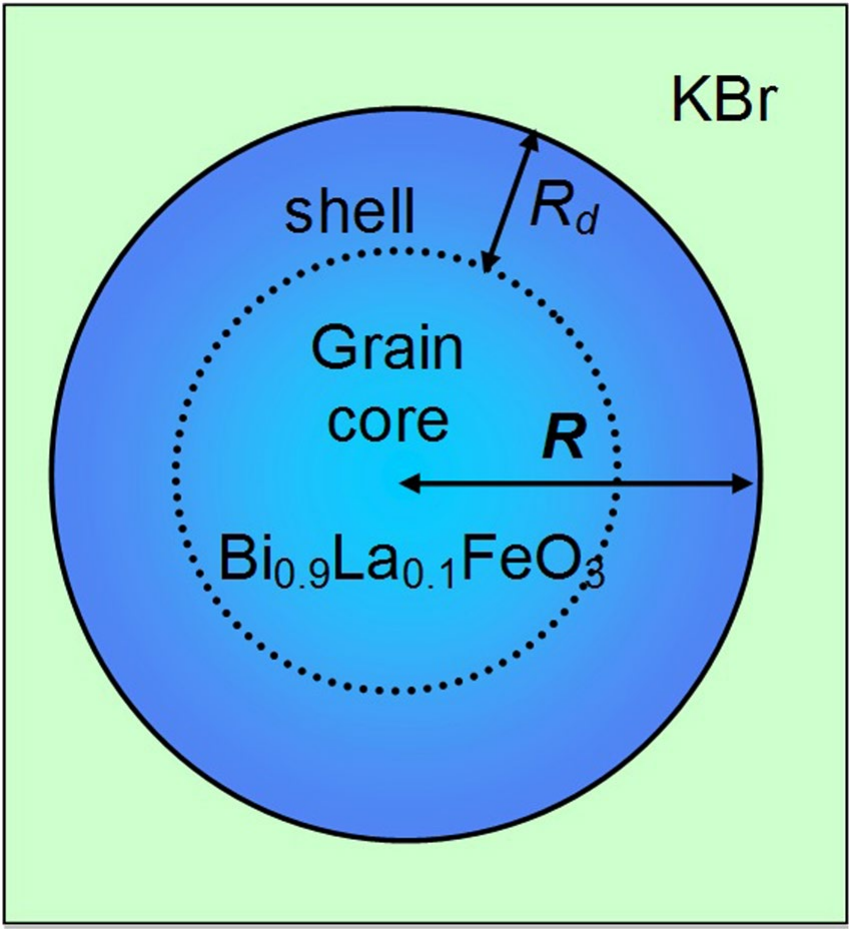
(**a**) Schematics of the spherical grain with radius *R* covered by the shell of thickness *R*_*d*_, where the defects are accumulated. Adapted from ref.^33^.

According to this, different physical mechanisms should be considered to explain the unexpected experimental result for 0.5(BLFO)–0.5(KBr) and 0.7(BLFO)–0.3(KBr) nanocomposites (the model is shown in Fig. 3), but only those among them, which are highly sensitive to the interaction of antiferromagnetic BLFO with ionic-conductor KBr, can be relevant.

Below we will try to prove that the appropriate mechanism turned out to be ferro-magneto-ionic coupling, that is a natural extension of ferro-ionic and anti-ferro-ionic couplings^48–51^ revealed earlier in ultra-thin ferroelectric films exposed to ionic exchange with ambient media (see original papers^52,53^, review^54^ and Refs therein). Following Stephenson and Highland (**SH**) model^52,53^ the screening by ions is electrically coupled to the electrochemical processes at the ferroelectric surface, and thus, the stabilization of ferroelectric state in ultrathin PbTiO_3_ films occurs due to the chemical switching^55–57^. The analysis^49,51^ leads to the elucidation of the ferro-ionic, anti-ferro-ionic and electret-like non-ferroelectric states, which are the result of nonlinear electrostatic interaction between the ferroelectric polarization and absorbed ions.

In accordance with experiments, the critical size of ferroelectricity and antiferromagnetism disappearance in BiFeO_3_ nanoparticles is very small – about 10 nm^58^. So that the “core” of 100-nm Bi_0.9_La_0.1_FeO_3_ particle for sure should be ferroelectric (**FE**) and anti-ferromagnetic (**AFM**). The ferromagnetism can be induced by the ferro-ionic exchange only in the vicinity of surface^59,60^, namely in a thin “shell” of thickness 2–5 nm (see Fig. 3).

In accordance with experimental results presented above (see Fig. 2), one can assume that in (BLFO)_x_–(KBr)_1-x_ nanocomposites the concentration of the “frustrated” spins in the shell of Bi_0.9_La_0.1_FeO_3_ nanoparticle first increases and then saturates with the increasing fraction of KBr. Thus, the idea of ferro-magneto-ionic coupling is that the surface of ferroelectric Bi_0.9_La_0.1_FeO_3_ is in the dynamic direct and indirect electron exchange with ionic conductor KBr. The role of the exchanges is twofold. The first one concerns the electronic states near the ferrite surface, which can lead to a rather weak ferromagnetic exchange due to the linear ME effect at the surface (as well as to DM-interaction, and/or geometric frustration). The mechanism will not be considered below.

The second is related to the fact that the interaction with nonmagnetic KBr matrix, via the rotostrictive and magnetostrictive coupling^35^, can induce the magnetic dipoles in the vicinity of surface of the Bi_0.9_La_0.1_FeO_3_ inclusions. At 25% concentration of ferromagnetic spins at the ferrite surface and under the surface, magnetic interaction between them in the particle “shell” cannot be excluded. In this case, the appearance of a loop with a relatively large coercive field occurs due to the mechanism of blocking the elementary spins.

### Theoretical modeling

To quantify the above idea, we use the thermodynamic potential of LGD-type that describes AFM, FE, and AFD properties of BFO nanoceramics, including the RM, RE and ME biquadratic couplings, and the AFD, FE, AFM contributions, as well as elastic energy in the form^33^.

Gibbs free energy of the nanoparticle (**NP**) has the following form:

1 $$G={G}_{M}+{G}_{ME},$$

where *G*_*M*_ and *G*_*ME*_ are the magnetic and magneto-elastic (magnetostriction and rotosriction) contributions to the NP energy, respectively.

The magnetic part of the free energy *G*_*M*_ describing the AFM-FM order-disorder type transition in the NP has the form:

2 $$\begin{array}{rcl}{G}_{M} & = & V(\frac{J}{2{N}_{d}}{\langle {l}_{i}\rangle }^{2}+\frac{{J}_{nl}}{4{N}_{d}}{\langle {l}_{i}\rangle }^{4}\\  &  & +\,\frac{{k}_{B}T}{2{N}_{d}}[(1+\langle {l}_{i}\rangle )\mathrm{ln}(1+\langle {l}_{i}\rangle )+(1-\langle {l}_{i}\rangle )\mathrm{ln}(1-\langle {l}_{i}\rangle )]\\  &  & -\,{M}_{S}\langle {l}_{i}\rangle {H}_{i}).\end{array}$$

The NP average volume is $V=\frac{4\pi }{3}{R}^{3}$. The dimensionless order parameter $\langle {l}_{i}\rangle$ is a degree of the magnetic dipoles ordering in the NP. Subscript *i* = 1, 2, 3 indicates Cartesian coordinates *x*, *y* and *z*. The value $\langle {l}_{i}\rangle$ is statistically averaged over the orientations of elementary magnetic dipoles *l*_*i*_ in the NP. The concentration *N*_*d*_ of magnetic dipoles is related with the concentration of ions at the NP surface. *J* is the exchange constant that is positive ($J\ge 0$) for the considered case of the AFM core, and related with Neel temperature *T*_*N*_ as $J={k}_{B}{T}_{N}$ in the mean field approximation (Boltzmann constant is ${k}_{B}=1.38\times {10}^{-23}\,{\rm{J}}/{\rm{K}}$). *J*_*nl*_ is the nonlinear exchange constant. The macroscopic magnetization components, ${M}_{i}={M}_{S}\langle {l}_{i}\rangle$, are coupled with the applied magnetic field *H*_*i*_.

The magneto-elastic contribution to the free energy (1) is:

3 $${G}_{ME}=-\,\langle {Z}_{klij}{{\rm{\sigma }}}_{kl}{M}_{S}^{2}{l}_{i}{l}_{j}+\frac{{s}_{ijkl}}{2}{{\rm{\sigma }}}_{ij}{{\rm{\sigma }}}_{kl}+{u}_{ij}^{W}{{\rm{\sigma }}}_{ij}\rangle .$$

In Equation (3) σ_*ij*_ is the elastic stress tensor, *Z*_*ijkl*_ is the magnetostriction stress tensor, and *s*_*ijkl*_ is the elastic compliances tensor of the AFM material. The summation is performed over all repeated indices. The bracket $\langle \ldots \rangle$ means the statistical averaging (summation) that is regarded equivalent to the intergation over the NP volume, ${\int }_{V}{d}^{3}r(\ldots )$ in the ergodic case. The last term in Eq. (3) is the Vegard-type energy density

4 $${u}_{ij}^{W}{{\rm{\sigma }}}_{ij}={{\rm{\sigma }}}_{ij}{W}_{ij}({\bf{r}}){\rm{\delta }}{N}_{S}({\bf{r}})\approx {z}_{ijkl}{W}_{ij}({\bf{r}}){\rm{\delta }}{N}_{S}({\bf{r}}){M}_{S}^{2}{l}_{k}{l}_{l}+o({l}_{k}^{2},{\rm{\delta }}{N}_{S}^{2}),$$

where we denote the elastic dipole tensor (Vegard expansion) of a surface defect as *W*_*ij*_^61^. Tensor ${z}_{ijkl}={Z}_{ijmn}{c}_{mnkl}$ is the magnetostriction strain tensor, *c*_*mnkl*_ is the elastic stiffness. The value ${\rm{\delta }}{N}_{S}({\bf{r}})\sim {\sum }_{k}{\rm{\delta }}({\bf{r}}-{{\bf{r}}}_{k})$ is the random concentration of elastic defects in the NP shell (including complexes with the surface ions and related with them). The approximate equality in Eq. (4) is valid if the main magnetization-dependent part of the stress is ${{\rm{\sigma }}}_{ij}^{M}={z}_{ijkl}{M}_{S}^{2}{l}_{k}{l}_{l}$ due to the magnetostriction mechanism. The function $o({l}_{k}^{2},{\rm{\delta }}{N}_{S}^{2})$ designates the small high order terms.

Allowing for the presence of the term ${{\rm{\sigma }}}_{ij}^{M}={z}_{ijkl}{M}_{S}^{2}{l}_{k}{l}_{l}$, the energy (3) changes the coefficient $\frac{J}{2{N}_{d}}$ in the term $\frac{J}{2{N}_{d}}{\langle {l}_{i}\rangle }^{2}$ in Eq. (2). Thus, the substitution of elastic fields (4) into the Eq. (3) and then to the Gibbs potential Eq. (1) leads to the renormalization of the coefficient $\frac{J}{2{N}_{d}}{\langle {l}_{i}\rangle }^{2}$ in Eq. (2), namely:

5 $$\begin{array}{rcl}\frac{J}{2{N}_{d}}{\langle {l}_{i}\rangle }^{2} & \to  & \frac{J}{2{N}_{d}}{\langle {l}_{i}\rangle }^{2}+\langle {z}_{kmii}{M}_{S}^{2}\sum _{n}{W}_{km}{\rm{\delta }}{N}_{S}({\bf{r}}-{{\bf{r}}}_{n}){l}_{i}{l}_{i}\rangle \\  & \approx  & (\frac{J}{2{N}_{d}}+{z}_{kmii}{M}_{S}^{2}\langle \sum _{n}{W}_{km}{\rm{\delta }}{N}_{S}({\bf{r}}-{{\bf{r}}}_{n})\rangle ){\langle {l}_{i}\rangle }^{2}.\end{array}$$

The statistical averaging over defect distribution in expression (5) gives:

6 $$\langle \sum _{n}{W}_{ij}{\rm{\delta }}{N}_{S}({\bf{r}}-{{\bf{r}}}_{n})\rangle \cong {W}_{ij}\frac{1}{V}{\int }_{V}{N}_{S}({\bf{r}})d{\bf{r}}.$$

The summation in Eq. (6) is performed over defect sites and the averaging of the function ${\rm{\delta }}{N}_{S}({\bf{r}}-{{\bf{r}}}_{l})$ in the equation leads to the integration over the shell region, where the defects are accumulated. For the clarity we assume that the distribution function of defects *N*_*S*_(**r**) depends on the distances from the NP surface $r=R$ (as the strongest inhomogeneity), and has exponential decay far from the surface [see Fig. 3]:

7 $${N}_{S}({\bf{r}})={N}_{S}(x)\exp (-\frac{R-r}{{R}_{d}}),\,0 < r < R$$

here ${R}_{d}\ll R$ is the decay length of defect concentration under the surface. The amplitude ${N}_{S}(x)$ depends on the KBr fraction (1-*x*) of NP surrounding, and preparation conditions of the composite. Indeed the ionic surrounding affects the defect formation energies, in accordance with e.g. Stephenson-Highland ionic adsorption model^50,53^. From Eq. (7), the average concentration of defects is

8 $${\bar{N}}_{S}(x,R)=\frac{1}{V}{\int }_{V}{N}_{S}({\bf{r}})d{\bf{r}}\approx 3{N}_{S}(x)(\frac{{R}_{d}}{R}).$$

The approximate equality is valid at ${R}_{d}\ll R$.

The temperature of the possible FM transition induced by surface ions can be defined from the expansion of the free energy (2) in the mean-field approximation, namely from the expansion up to quadratic terms of the expression

9a $$\begin{array}{l}[\frac{J}{2{N}_{d}}+{z}_{kmii}{W}_{km}{\bar{N}}_{S}{M}_{S}^{2}]{\langle {l}_{i}\rangle }^{2}+\frac{{J}_{nl}}{4{N}_{d}}{\langle {l}_{i}\rangle }^{4}\\ \,+\frac{{k}_{B}T}{2{N}_{d}}[(1+\langle {l}_{i}\rangle )\mathrm{ln}(1+\langle {l}_{i}\rangle )+(1-\langle {l}_{i}\rangle )\mathrm{ln}(1-\langle {l}_{i}\rangle )]\\ \,\approx \frac{{{\rm{\alpha }}}_{i}}{2}{\langle {l}_{i}\rangle }^{2}+\frac{{\rm{\beta }}}{4}{\langle {l}_{i}\rangle }^{4}+\mathrm{..}O{\langle {l}_{i}\rangle }^{5}\end{array},$$

The coefficients α and β have the following form:

9b $$\begin{array}{rcl}{{\rm{\alpha }}}_{i}(x,R,T) & = & \frac{J}{{N}_{d}}+2{z}_{kmii}{W}_{km}{\bar{N}}_{S}(x,R){M}_{S}^{2}+\frac{{k}_{B}T}{{N}_{d}},\\ {\rm{\beta }}(T) & = & (\frac{{k}_{B}T}{3}-{J}_{nl})\frac{1}{{N}_{d}}.\end{array}$$

The critical temperature ${T}_{cr}^{i}(x,R)$ satisfies the equation ${{\rm{\alpha }}}_{i}(x,R,T)=0$ and acquires the form:

10 $${T}_{cr}^{i}(x,R)=-\frac{J+2{z}_{kmii}{W}_{km}{\bar{N}}_{S}(x,R){N}_{d}{M}_{S}^{2}}{{k}_{B}}.$$

Since $J > 0$, the product ${z}_{kmii}{W}_{km}{\bar{N}}_{S}(x,R){N}_{d}$ should be negative in order to make ${T}_{cr}(x,R)$ positive under the condition $J+2{z}_{kmii}{W}_{km}{\bar{N}}_{S}(x,R){N}_{d}{M}_{S}^{2} < 0$. Hereinafter we assume that the Vegard tensor is isotropic and diagonal, i.e. ${W}_{ij}=W{{\rm{\delta }}}_{ij}$, and magnetostriction tensor symmetry is cubic, i.e. ${z}_{1111}={z}_{2222}={z}_{2222}\equiv z$. So that ${z}_{kmii}{W}_{km}\equiv z\cdot W$. We should underline that positive ${T}_{cr}(x,R)$ can originate from magnetostivie coupling.

For a semi-quantitative description of the magnetization curves observed in our experiments [see Fig. 2] we model the dynamics of magnetization dependence on the quasi-static magnetic field from the relaxation time-dependent equation

11 $${\rm{\Gamma }}\frac{dl}{dt}=(\frac{J}{{N}_{d}}+2zW{M}_{S}^{2}{\bar{N}}_{S})l+\frac{{J}_{nl}}{{N}_{d}}{l}^{3}+\frac{{k}_{B}T}{2{N}_{d}}\,\mathrm{ln}(\frac{1+l}{1-l})-H{M}_{S}.$$

Hereinafter ${\rm{\Gamma }}$ is the relaxation coefficient. In the static case the right-hand side of Eq. (11) can be expanded in *l*-series as ${\rm{\alpha }}l+{\rm{\beta }}{l}^{3}-H{M}_{S}$. The values of the remanent magnetization and “intrinsic” thermodynamic coercive field *H*_*c*_ can be estimated from the expansion as:

12 $${M}_{r}(x,R,T)\approx \sqrt{-\frac{{\rm{\alpha }}}{{\rm{\beta }}}},\,{H}_{C}({T}_{r},h)={\rm{\alpha }}\sqrt{\frac{27{\rm{\alpha }}}{4{\rm{\beta }}}}.$$

**Figure 4:**
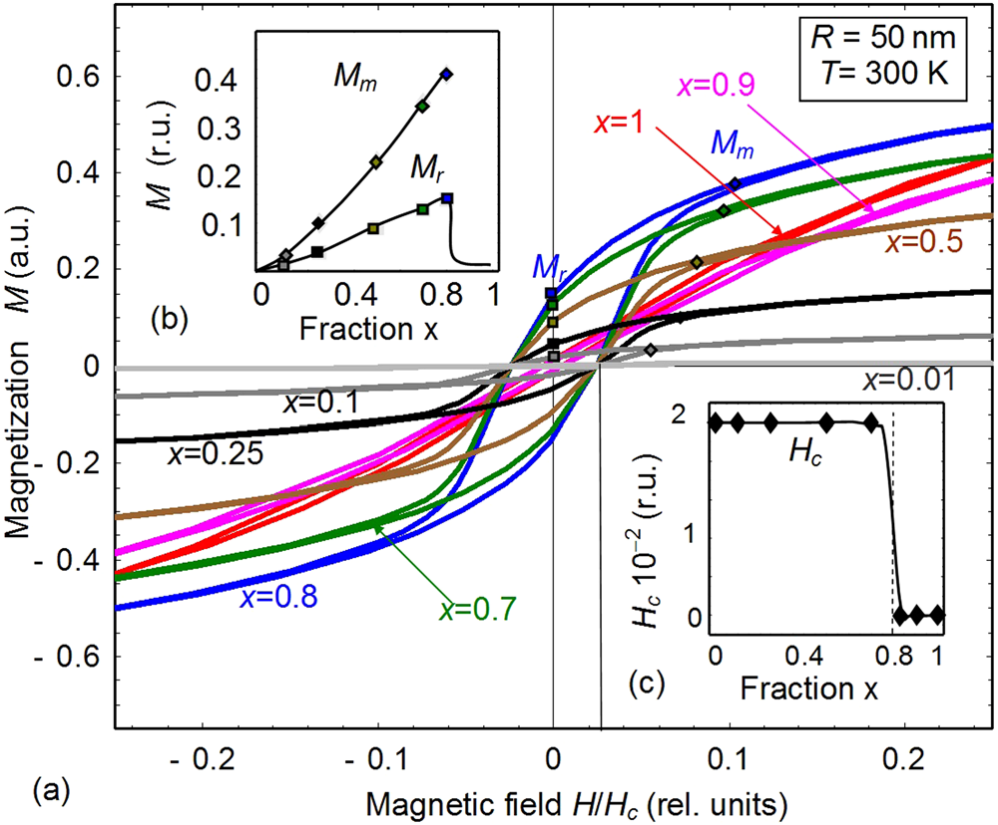
(**a**) Magnetization dependence on quasi-static magnetic field calculated for (Bi_0.9_La_0.1_FeO_3_)_x_ – (KBr)_1-x_ nanocomposite at RT. Different curves (1–7) correspond to the gradual decrease of the Bi_0.9_La_0.1_FeO_3_ fraction x = 1, 0.9, 0.8, 0.7, 0.5, 0.25, 0.1 and 0.01 (see labels near the curves). Two insets (**b**,**c**) show the dependence of the remanent magnetization (M_r_), maximal in-field magnetization (M_m_) and coercive field (H_c_) on the fraction x of KBr. The average radius *R* of Bi_0.9_La_0.1_FeO_3_ nanoparticles was 50 nm, parameters *J*/*k*_*B*_ = −650 K, *z* = 5 × 10^−3^ magnetic units, ${\bar{N}}_{S}$ = (1.4 − 1.6) × 10^23^ m^−3^ for the curves (1–7), respectively, *W* = −10 Å^3^, and *R*_*d*_ = 2 nm.

The dependence of magnetization of (BLFO_3_)_x_–(KBr)_1-x_ nanocomposite on quasi-static magnetic field was calculated from Eq. (11) at RT. Results are presented in Fig. 4(a). Different curves correspond to the gradual decrease of the BLFO fraction x = 1, 0.9, 0.8, 0.7, 0.5, 0.25, 0.1 and 0.01 (see labels near the curves). Two insets Fig. 4(b,c) illustrate the dependence of the remanent magnetization (M_r_), maximal in-field magnetization (M_m_) and coercive field (H_c_) on the fraction 1-x of KBr.

There are evident qualitative similarities (such as the loop order and shape changes with *x* increase) between Figs 2 and 4. However they differ quantitatively. In particular, the calculated concentration dependence of the remanent magnetization and the saturation law at high magnetic fields significantly differs from the experiment. It appears that the experimental data shown in Fig. 2 can be rigorously fitted (with not more than several % error) by the function

13 $$M(H)={M}_{0}\,\tanh (\frac{H-{H}_{S}}{{\rm{\Delta }}})-\chi H-{M}_{H}$$

Here *M* is the magnetization, *H* is the applied magnetic field. The fitting parameters *M*_0_, *H*_*S*_, Δ, $\chi$ and *M*_*H*_ depend on BLFO concentration *x* and are listed in Table S-1 in the Supplement. They are different for the “up” and “down” parts of the loops, and the small values of *M*_*H*_ can be regarded as the error. Notably that Eq. (13) contains a small linear term $\chi H$ not included in the theoretical model (11), which magnitude depends on x% of BLFO. The presence of the linear term may be associated with the net response of the Bi_0.9_La_0.1_FeO_3_ ceramics, which magnetization quasi-linearly increases with *H*. The presence of the nonlinear term ${M}_{0}\,\tanh (\frac{H-{H}_{S}}{{\rm{\Delta }}})$ corroborates the validity of the Ising-type order-disorder Eq. (11) used for the qualitative understanding of the experimental results. Actually, the approximate analytical solution of the static equation (11) is $l=\,\tanh (\frac{H{M}_{S}{N}_{d}}{{k}_{B}T})$ that is rigorous at negligibly small contributions of the linear and nonlinear terms $(\frac{J}{{N}_{d}}+2{z}_{ijkk}{W}_{ij}{\bar{N}}_{S}{M}_{S}^{2})l$ and $\frac{{J}_{nl}}{{N}_{d}}{l}^{3}$, respectively. The nonlinear contribution to the magnetostatic response is attributed with ferro-magneto-ionic coupling between BLFO and KBr, mediated by magnetostriction.

## Conclusion

We studied magnetostatic response of the composites consisting of nanosized ferrite Bi_0.9_La_0.1_FeO_3_ conjugated with fine grinded ionic conducting KBr powder. When the fraction of KBr is rather small (less than 15%) the magnetic response of the composite is very weak and similar to that observed for the compound Bi_0.9_La_0.1_FeO_3_; pure KBr matrix without Bi_1-x_La_x_FeO_3_ has no magnetic response at all as anticipated. However, when the fraction of KBr increases above 15%, the magnetic response of the composite changes substantially and the field dependence of magnetization discloses ferromagnetic-like hysteresis loops with the remanent magnetization about 0.14 emu/g and the coercive field about 1.8 Tesla (at room temperature). Nothing similar to the ferromagnetic-like hysteresis loop can be observed in Bi_0.9_La_0.1_FeO_3_ ceramics, which magnetization quasi-linearly increases with magnetic field.

Different physical mechanisms were proposed to explain the unusual experimental results for nanocomposites Bi_0.9_La_0.1_FeO_3_-KBr, but only those among them, which are highly sensitive to the interaction of antiferromagnetic Bi_0.9_La_0.1_FeO_3_ with ionic conductor KBr, can be relevant. We have shown that the ferromagnetic behavior appears as a synergetic effect driven by the ferro-magneto-ionic and magnetostrictive coupling.

## Supplementary information

Supplement
