# Supplementary material for: Ferromagnetic-like behavior of Bi0.9La0.1FeO3–KBr nanocomposites
Source: Sci Rep. 2019 Jul 18;9:10417. doi: 10.1038/s41598-019-46834-0 (PMC6639540; doi:10.1038/s41598-019-46834-0)
Supplement: Supplementary file 1 — Supplement [file 41598_2019_46834_MOESM1_ESM.pdf]

Ferromagnetic-like behavior of $Bi_{0.9}La_{0.1}FeO_3$ – KBr nanocomposites

Dmitry V. Karpinsky, Olena M. Fesenko, Maxim V. Silibin, Sergei V. Dubkov, Mykola Chaika, Andrii Yaremkevich, Anna Lukowiak, Yuri Gerasymchuk, Wiesław Stręk, Andrius Pakalniškis, Ramunas Skaudzius, Aivaras Kareiva, Yevhen M. Fomichov, Vladimir V. Shvartsman, Sergei V. Kalinin, Nicholas V. Morozovsky, and Anna N. Morozovska

SUPPLEMENT

SAMPLES CHARACTERIZATION

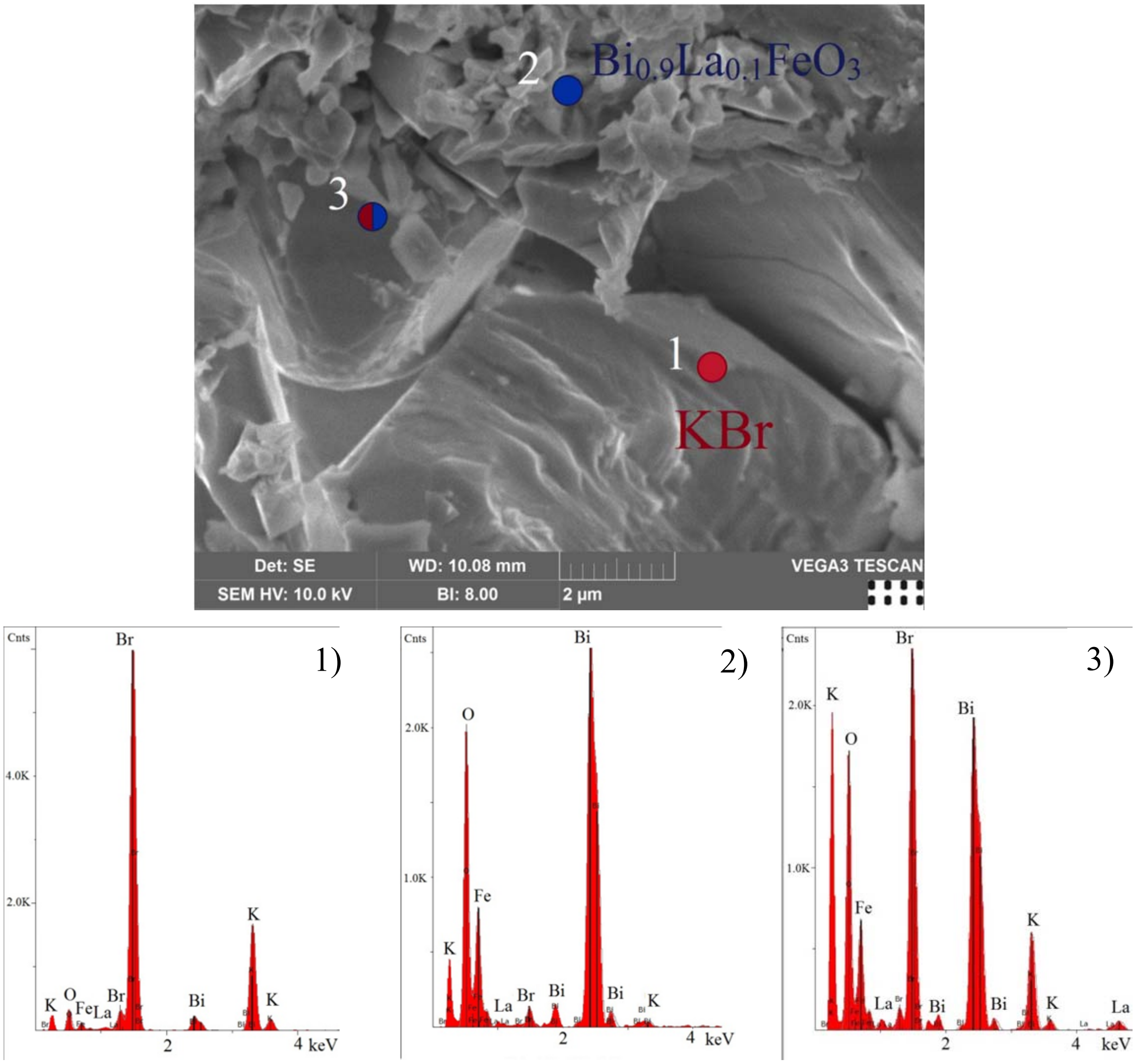

Figure S1. SEM image of the composite $Bi_{0.9}La_{0.1}FeO_3$ - KBr (50:50) and 1) - 3) EDS data calculated for the selected areas of the composite as marked at SEM image.

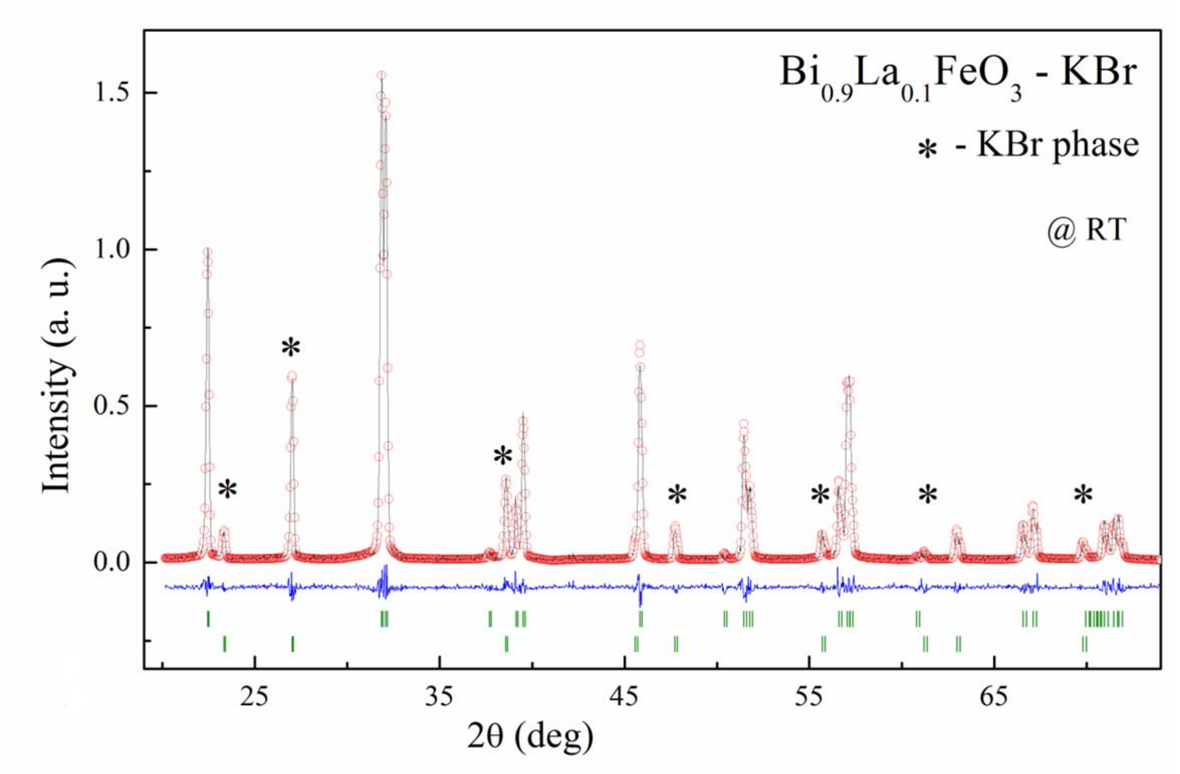

Figure S2. Refined XRD pattern obtained for the composite $(Bi_{0.9}La_{0.1}FeO_3)_{0.8}$–$(KBr)_{0.2}$ at room temperature (circles are experimental data, lines are calculated ones). Upper row of the Bragg reflections are associated with the ferrite phase (space group R3c), KBr phase is denoted by asterisks.

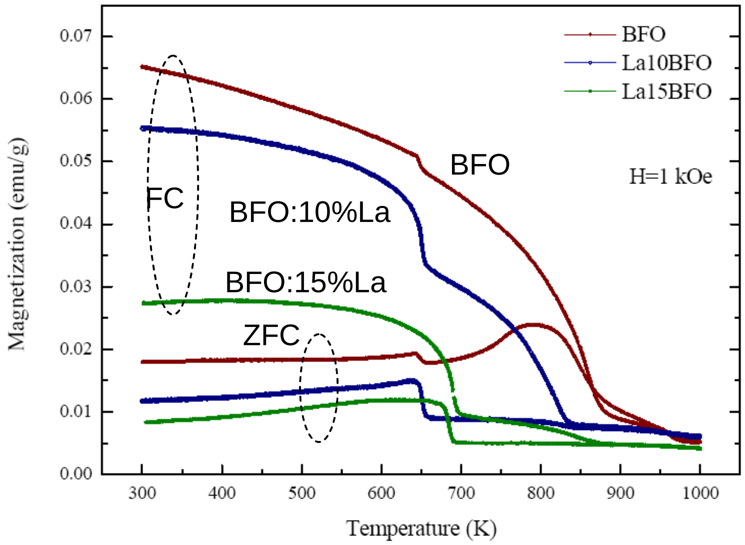

Figure S3. Temperature dependencies of $Bi_{1-z}La_zFeO_3$ magnetization (z=0, 0.1 and 0.15) measured using MPMS SQUID VSM magnetometer in the field cooled (brown, dark blue and green upper curves "FC") and zero field cooled (brown, dark blue and green lower curves "ZFC") modes in a static magnetic field of 1kOe in the temperature range 300 – 1000 K.

For the sake of comparison with the composite, we studied how the increase of La content affects the field dependencies of magnetic moment M(H) of the AFM compounds $Bi_{1-z}La_zFeO_3$ for x = (0 - 0.17). Isothermal dependencies of magnetization M(H) shown in **Fig. S4** demonstrate complex behavior of M(H) curves in strong magnetic fields which changes with the dopant content. The compounds with the dopant content $z \leq 0.13$ are characterized by the M(H) dependencies which show metamagnetic transition behavior associated with a disruption of spatially modulated magnetic structure (specific for initial compound $BiFeO_3$).

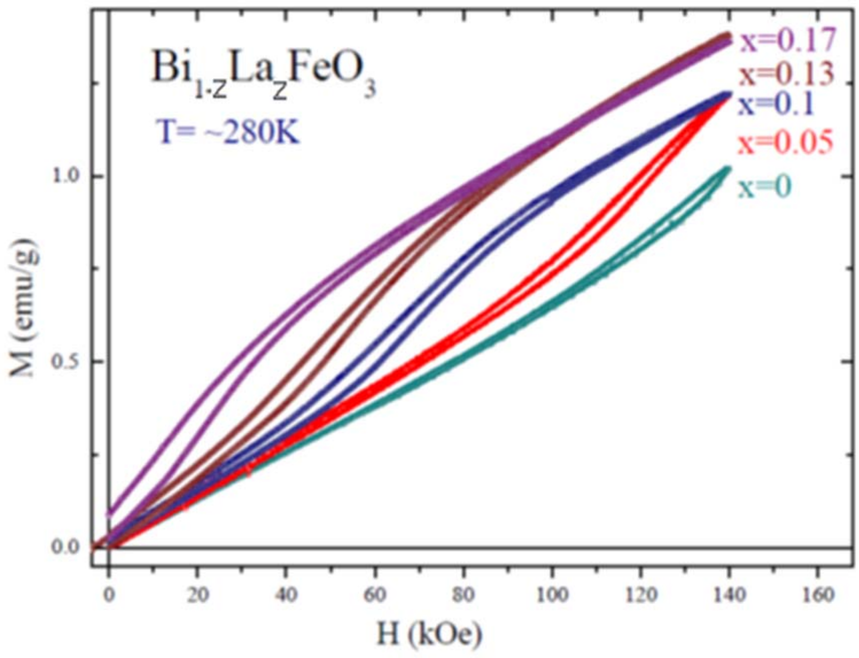

Figure S4. M(H) dependencies measured at room temperature for compounds $Bi_{1-z}La_zFeO_3$, where z = 0, 0.05, 0.1, 0.13 and 0.17.

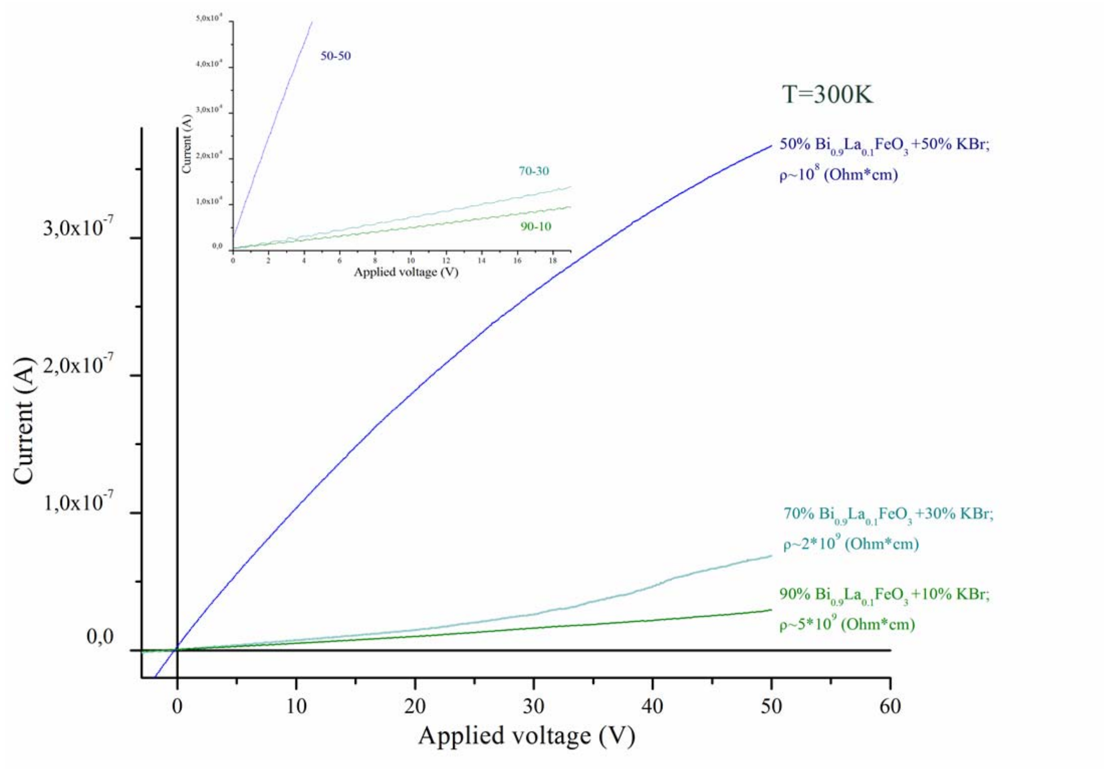

Figure S5. Volt-ampere characteristics of the composites (x)$Bi_{0.9}La_{0.1}FeO_3$ – (1-x)KBr (x=0.1, 0.3, 0.5). The inset shows enlarged area of the I(V) dependencies at low applied voltages.

Volt-ampere characteristics measured in ***dc*** mode for the composites $Bi_{0.9}La_{0.1}FeO_3$ – KBr, are shown in **Fig. S5.**

Table S-I. Fitting parameters in Eq. (13)

**x (%) (BLFO)**	M_0	H_S	Δ	χ	M_H
**50 (up)**	-0,0917	-2,4883	-2,0111	-0,0276	-0,0036
**50 (down)**	-0,0961	2,5029	-2,0883	-0,0272	0,0018
**70 (up)**	-0,1492	-2,7157	-2,0508	-0,0490	-0,0066
**70 (down)**	-0,1582	2,7932	-2,1669	-0,0484	0,0006
**80 (up)**	-0,1565	-3,3735	-2,4499	-0,0527	-0,0055
**80 (down)**	-0,1666	3,4416	-2,6627	-0,0522	0,0005
**85 (up)**	-0,0226	-6,6238	-0,4442	-0,0713	0,0054
**85 (down)**	-0,0236	6,3713	-0,00645	-0,0713	-0,0067
**90 (up)**	-0,0303	-6,7996	-0,3464	-0,0980	0,0070
**90 (down)**	-0,0386	6,8332	-0,4350	-0,0970	-0,0166
**100 (up)**	-0,0348	-6,3419	-0,4935	-0,0984	0,00126
**100 (down)**	-0,0427	6,3601	-0,6061	-0,0975	-0,0108
